# Active cycle of breathing technique may reduce pulmonary complications after esophagectomy: A randomized clinical trial

**DOI:** 10.1111/1759-7714.14227

**Published:** 2021-11-12

**Authors:** Jiudi Zhong, Siwen Zhang, Chuangzhen Li, Yi Hu, Weijin Wei, Li Liu, Ming Wang, Zhangxian Hong, Hao Long, Tiehua Rong, Hong Yang, Xiaodong Su

**Affiliations:** ^1^ Department of Thoracic Surgery Sun Yat Sen University Cancer Center, Guangzhou; State Key Laboratory of Oncology in Southern China and Collaborative Innovation Center for Cancer Medicine Guangzhou China

**Keywords:** active cycle of breathing technique, esophageal carcinoma, esophagectomy, pulmonary complication

## Abstract

**Background:**

The purpose of the study was to determine whether the active cycle of breathing technique (ACBT) has an impact on postoperative pulmonary complication (PPC) after esophagectomy.

**Methods:**

In this prospective randomized trial, patients who were candidates for esophagectomy were randomized into groups, wherein they received either ACBT (*n* = 146) or conventional chest physiotherapy (control group, *n* = 145) on postoperative days (POD) 1–3. The primary outcome was PPC. The secondary outcomes included the incidence of anastomotic leakage (AL), efficacy of airway clearance, and postoperative hospital length of stay (LOS).

**Results:**

After esophagectomy, the PPC rate was significantly lower in the ACBT group (15.2%) than in the control group (31.0%) (*p* = 0.001). The incidences of AL were 5.5% and 12.4% in the ACBT and control groups, respectively (*p* = 0.042). Mean hospital LOS was 12.3 days for the ACBT group and 16.8 days for the control group (*p* = 0.008). ACBT significantly increased the mean sputum wet weight (g) on POD 1–3 when compared with conventional therapy (POD 1 9.08 vs. 6.47, POD 2 16.86 vs. 10.92, POD 3 24.65 vs. 13.52, all *p* < 0.001). Multivariable analysis revealed that ACBT decreased the rates of PPC (odds ratio [OR] 0.403, *p* = 0.003), AL (OR 0.379，*p* = 0.038)，arrhythmia (OR 0.397, *p* = 0.028), and bronchoscopy aspiration (OR 0.362, *p* = 0.016).

**Conclusion:**

ACBT is an effective airway clearance technique that significantly reduces the incidence of PPC after esophagectomy. ACBT could also significantly reduce both AL and LOS.

## Graphical abstract

The effect of the active cycle of breathing technique (ACBT) on postoperative pulmonary complication (PPC) after esophagectomy has not yet been investigated. In this randomized clinical trial that included 291 patients with resectable thoracic esophageal carcinoma, the PPC rate was significantly lower in the ACBT group (15.2%) than in the control group (31.0%) (*p* = 0.001) after esophagectomy.

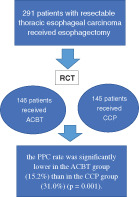

## INTRODUCTION

Esophageal carcinoma (EC) is one of the most common malignant tumors, and it was estimated that approximately 246 000 new cases occur annually in China.^1^ Surgical resection is the preferred curative treatment for localized EC. Despite improvements in surgical techniques and perioperative care, esophagectomy remains a complex procedure requiring extended operative time. In addition, anesthesia causes postoperative pathophysiological reduction in lung volume, respiratory muscle function, and mucociliary clearance and an increase in the retention of secretion.^2^ Postoperative pulmonary complication (PPC) is the most common type of complication observed after esophagectomy; the incidence of PPC ranges from 20% to 37%.^3^, ^4^, ^5^, ^6^, ^7^, ^8^ PPC has been reported to be associated with a considerable increase in morbidity and mortality,^4^, ^5^, ^6^ risk of anastomotic leakage (AL),^9^ postoperative hospital length of stay (LOS), and healthcare cost.^10^

Atelectasis and pneumonia are the most frequent PPCs that occur after thoracic surgery,^2^ and they are usually caused and worsened by retention of secretion.^11^, ^12^ Chest physiotherapy for airway clearance after thoracic surgery is recommended to improve reduced lung volume, assist secretion clearance, and improve mobility, thus reducing the risk of developing PPC.^13^ Conventional chest physiotherapy (CCP) involves deep breathing exercises and manual chest percussion (clapping) to assist patients in clearing sputum from their airways.^14^ However, CCP is labor‐intensive and can potentially cause patient discomfort.^15^ Patients are reluctant to cough deeply and are incapable of effectively clearing their airway secretions by themselves.

Active cycle of breathing technique (ACBT) is an alternative airway clearance technique. A typical ACBT cycle consists of breathing control, three to four thoracic expansion exercises, and a forced expiratory technique (huffing).^14^ ACBT has been shown to improve short‐term secretion clearance in patients with chronic lung disease.^16^ It is also flexible, tolerated, and accepted well by patients.^14^, ^16^ Although ACBT is widely used in patients with respiratory conditions characterized by chronic sputum production, such as cystic fibrosis and bronchiectasis,^17^ current literature on perioperative chest physiotherapy involving ACBT after thoracic surgery is limited. A quasi‐experimental study found that ACBT could improve secretion removal and functional exercise capacity for lung cancer patients after lobectomy, but it did not significantly decrease PPC.^18^ The objective of this study was to investigate the impact of ACBT on PPC after esophagectomy. The primary outcome assessed was PPC. Secondary outcomes included incidence of AL, efficacy of airway clearance, and postoperative hospital LOS.

## METHODS

### Study design

This randomized single‐center clinical trial was conducted in Sun Yat‐sen University Cancer Center (SYSUCC) between December 2017 and August 2019. The study protocol was approved by the SYSUCC Ethics Committee (approval number: GYX2017‐003). All enrolled patients signed an informed consent form.

### Participants

Preoperative evaluation included endoscopic ultrasonography with biopsy, computerized tomography scan of the chest and abdomen, and ultrasonography of the neck. Eligible patients included those with histologically confirmed squamous cell carcinoma, a resectable disease (cT1‐3,N0‐1 and M0). Exclusion criteria included history of other malignant tumors, administration of neoadjuvant radiotherapy or chemotherapy, unwillingness or inability to participate in chest physiotherapy, critical condition or death after operation, and cognitive impairment.

### Randomization

Eligible patients were randomly assigned to receive either CCP or ACBT when admitted to the hospital. Randomization was performed using the sealed envelope method. Sequentially numbered sealed envelopes, containing intervention grouping information, were prepared and provided by the Department of Biostatistics of SYSUCC. The two assessors who collected the outcome data were unaware of the assignment information.

### Surgery

All operations were performed by experienced thoracic surgeons. The surgical procedure consisted of Sweet, Ivor‐Lewis, or McKeown esophagectomy, determined by the location of the tumor, extent of the disease, and surgeon's preference. The surgical approach was either open thoracotomy or minimally invasive esophagectomy. Gastric tube reconstruction was performed using linear staplers, and the conduit was brought into the thoracic cavity through the posterior mediastinal route (Sweet or Ivor‐Lewis) or up to the neck through the posterior sternum route (McKeown). Mediastinal lymphadenectomy was routinely performed. Patients received opioid analgesics subcutaneous injection if necessary after operation.

### Chest physiotherapy intervention

CCP (control group) involves deep breathing exercises and manual chest percussion (clapping). The thoracic surgical nurses instructed participants to perform deep breathing exercises and effective coughing on the day of admission and the day before operation in the ward. CCP was performed by a thoracic surgical nurse four times daily on postoperative days (POD) 1 to 3. Patients received manual clapping over the chest wall and the back at a frequency of more than 100 times/min in the semi‐recumbent or sitting position. After percussion, the patients took deep breaths, coughed, and expectorated. Each session lasted for 10–15 min.

A complete ACBT consisted of three to five breath control sessions, three to four chest expansion exercises, and two to three forced expiratory techniques. The number and frequency of each ACBT component can be altered, but all components of the cycle must be present and interspersed with breathing control.^16^, ^18^ The patients received a booklet with instructions on how to perform ACBT as well as instruction by a thoracic surgical nurse on the day of admission. A total of six interventions were conducted in the ward on the first and second days after admission and the day before the operation. Because the patients retained the neck drainage tube and the thoracic drainage tube after the operation, they received the intervention at bedside once daily on POD 1–3. Each intervention lasted for about 10–15 min.

Patients assumed the sitting or supine position and relaxed their shoulders before intervention. The method of respiratory control was as follows: inhale deeply and slowly through the nose three times, hold for 3 s after the last inhalation, then perform a moderate‐ to low‐degree lip‐contracting exhalation to achieve an inspiration‐expiration ratio of 1:2–3; this is performed consecutively three to five times to clean up the surrounding respiratory secretions. Chest expansion training was performed as follows: hold for 3 s after active deep inspiration, feel the expansion of the thorax, then passively relax and exhale three to four times, and vibrate the secretions by contracting chest wall muscles. The forced expiratory technique was performed as follows: when the secretion reaches the central airway, inhale deeply, then actively and forcefully retrieve the abdomen with force, open the glottis at the same time, and emit two to three low‐level breaths (forced sighs) when exhaling. Repeat deep inhalation, exhale forcefully to emit two to three strong breaths. Rrepeat this two to three times in a row, and then breathe in a controlled manner. After completing the above actions, the patients were asked to cough up the residual deep sputum to promote pulmonary expansion. Patients were encouraged to repeat three to five cycles of training for 10–15 min per cycle and complete at least four ACBT training sessions daily.

### Outcome measures

The outcomes were recorded by two assessors who were blinded to the intervention. The primary outcome was incidence of PPC during POD in the hospital. Patients were screened using the Melbourne Group Score (MGS), a standardized validated diagnostic tool consisting of eight symptomatic and diagnostic criteria.^19^ PPC was diagnosed when four or more of the following eight criteria were present: chest X‐ray findings of atelectasis/consolidation, unexplained increased white cell count (>11.2 × 109/L) or administration of respiratory antibiotics, fever >38°C, signs of infection in sputum microbiology, purulent sputum (yellow or green) differing from the preoperative status, oxygen saturation <90% at room air, physician's diagnosis of pneumonia or chest infection, and readmission to the intensive care unit (ICU) for respiratory complications or prolonged stay (>36 h) in the ICU.

The secondary outcomes included the following: (1) AL, defined as the clinical presentation of vast secretions in the cervical incision or an anastomotic disruption detected by X‐ray contrast examination and confirmed by upper endoscopy; (2) efficacy and acceptability of techniques for airway clearance: (a) sputum wet weight (SWW) (g), a common and clinical useful outcome of airway clearance techniques.^16^ Sputum was collected in a clear sterile pot (mean weight 6.5 g/pot) during and up to 24 h before operative day (BOD) 1 and POD 1–3. The total expectorated sputum was weighed using a calibrated electronic balance (New Health, Model No: 20161206, manufactured 2016), accurate to 0.01 g; (b) peak expiratory flow (PEF), a parameter considered to reflect large airway function and commonly serving as a global indicator of airway function;^20^ (c) patient comfort during chest physiotherapy, assessed using a visual analog scale (VAS) containing a horizontal line 10 cm long, with markings from 1 to 10, with 1 indicating that the patients are comfortable with the therapy and 10 indicating intolerable pain. The patients were required to provide a patient's comfort response immediately after every treatment, either CCP or ACBT, and the average of the comfort scores was reported.

Other outcomes included arrhythmia, defined as persistent supraventricular tachycardia requiring anti‐arrhythmia agent treatment, and arterial blood gas analysis was performed on POD 5 by measuring and recording partial arterial oxygen (PaO_2_) pressure.

### Statistical analysis

A power analysis was performed and sample size software was used to calculate the sample size. Previous data indicated chest physiotherapy may reduce 40–70% of PPC in cardio‐thoracic surgery.^21^, ^22^, ^23^ To the supposed reduction of 40% of the PPC, with an estimation of 15% dropout and 80% statistical power, a sample size of 148 patients per group was necessary.

Continuous data were expressed as mean and median values, with discrete variables expressed as frequencies. For bivariable analyses, Mann–Whitney tests were used to compare continuous data and the Fisher exact or χ^2^ test for categorical variables. Multivariable regression analysis was used to control for known confounders of postoperative morbidity, such as age, smoking, American Society of Anesthesiologists (ASA) classification of at least 3, diabetes, hypertension, and preoperative weight loss of 10% or more. All statistical analyses were performed using the Statistical Package for the Social Sciences (SPSS) v22.0.

## RESULTS

### Patient characteristics

**FIGURE 1:**
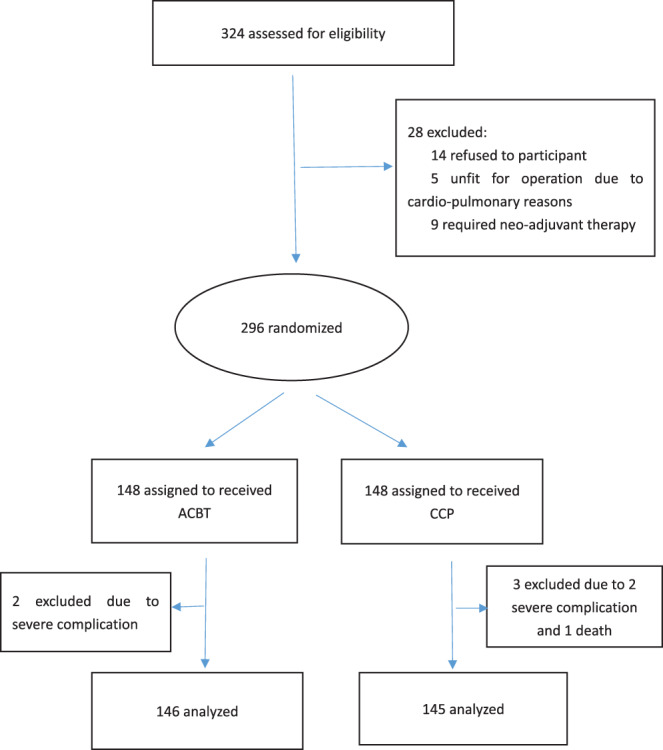
Flow diagram of the study participants

Between December 2017 and August 2019, 291 eligible candidates for esophagectomy were randomly assigned to receive either CCP (control group, *n* = 145) or ACBT (*n* = 146) (Figure 1). Baseline demographics and clinical characteristic such as age, sex, smoking index, body mass index, comorbid conditions (diabetes mellitus, hypertension, coronary artery disease), ASA score, tumor location, and procedure of esophagectomy of the two groups were similar between the groups (Table 1).

**TABLE 1:** Patient characteristics

Characteristic		Patients	*n* (%)	*p* value
		ACBT (*n* = 146)	Control (*n* = 145)	
Age (years), mean (SD)		61.2(8.61)	61.1(8.25)	0.995
Sex (male/female)		108(74.0)/38(26.0)	111(76.6)/34(23.4)	0.684
Smoking index ≥400		79(54.1)	77(53.1)	0.907
Body mass index ≥18.5 kg/m^2^		128(87.7)	130(89.7)	0.712
Weight loss >10%		6(4.1)	10(6.9)	0.318
Comorbid condition				
Hypertension		26(17.8)	26(17.9)	1.000
Coronary artery disease		1(0.7)	0(0)	1.000
Diabetes mellitus		17(11.7)	11(7.6)	0.320
ASA score	1–2	126(86.3)	124(85.5)	0.868
	3	20(13.7)	21(14.5)	
FEV1 (L), mean (SD)		2.58(0.66)	2.57(0.61)	0.992
Tumor location				
Upper thoracic		11(7.5)	13(9.0)	0.225
Middle thoracic		69(47.3)	81(55.9)	
Lower thoracic		66(45.2)	51(35.1)	
Procedure				
Sweet		9(6.2)	9(6.1)	0.980
Ivor‐Lewis		54(37.0)	52(35.9)	
McKeown		83(56.8)	84(57.9)	
Approach				
Open thoracotomy		82(56.2)	79(54.5)	0.814
MIE		64(43.8)	66(45.5)	
Site of anastomosis				
Cervical		83(56.8)	84(57.9)	0.906
Intra‐thoracic		63(43.2)	61(42.1)	
Operative time				
<3 h		32(21.9)	34(23.4)	0.781
≥3 h		114(78.1)	111(76.6)	

Abbreviations: ACBT, active cycle of breathing technique; ASA, American Society of Anesthesiologist; FEV1, forced expiratory volume in 1 s; MIE, minimally invasive esophagectomy; SD, standard deviation.

### Morbidity

The PPC incidence after esophagectomy in the ACBT group (15.2%) was significantly lower than that in the control group (31.0%) (*p* = 0.001). Furthermore, patients in the ACBT group had a significant reduction in both AL incidence (ACBT 5.5% vs. control 12.4%, *p* = 0.042) and arrhythmia (ACBT 8.2% vs. control 15.9%, *p* = 0.049) (Table 2). The ACBT group had a significantly shorter mean postoperative LOS than the control group (12.3 days vs. 16.8 days, *p* = 0.009). After adjusting for potential cofounders, multivariable analysis showed that ACBT decreases the odds of PPC (odds ratio [OR] 0.403, *p* = 0.003), AL (OR 0.379, *p* = 0.038), and arrhythmia (OR 0.397, *p* = 0.028) (Table 3).

**TABLE 2:** Outcomes after esophagectomy

Outcome	Patients	*n* (%)	*p* value
	ACBT (*n* = 146)	Control (*n* = 145)	
PPC	22(15.2)	45(31.0)	0.001
Pneumonia	6(4.1)	15(10.3)	0.044
Atelectasis	4(2.7)	9(6.2)	0.169
Hypoxia	8(5.5)	18(12.4)	0.042
ARDS	2(1.4)	6(4.1)	0.173
Anastomotic leakage	8(5.5)	18(12.4)	0.042
Arrhythmia	12(8.2)	23(15.9)	0.049
Chylothorax	2(1.4)	2(1.4)	1.000
Length of stay (days), mean (SD)	12.3(11.3)	16.8(15.4)	0.009

Abbreviations: ACBT, active cycle of breathing technique; ARDS, acute respiratory distress syndrome; PPC, postoperative pulmonary complication.

**TABLE 3:** Adjusted odds ratio of morbidity due to ACBT

Characteristic	Adjusted odds ratio (95% CI)^a^	*p* value
PPC	0.403(0.222–0.734)	0.003
Anastomotic leakage	0.379(0.172–0.948)	0.038
Arrhythmia	0.397(0.173–0.903)	0.028
Bronchoscopy aspiration	0.362(0.158–0.827)	0.016

Abbreviations: ACBT, active cycle of breathing technique; CI, confidential interval; PPC, postoperative pulmonary complication.

^a^ Odds ratios were decreased in the ACBT group and were adjusted for age, smoking status, comorbid conditions, American Society of Anesthesiologists score ≥ 3, and weight loss >10%.

### Efficacy and acceptability of airway clearance techniques

The mean SWWs (g) of the ACBT and control groups on BOD 1 were 2.30 and 2.32, respectively (*p* = 0.705). The mean daily SWW significantly increased in the ACBT group as compared to that in the control group on POD 1–3 (POD 1 9.60 vs. 6.88, POD 2 16.93 vs. 11.28, POD 3 24.11 vs. 14.06, all *p* < 0.001). The mean PEFs (L/min) of the ACBT and control groups before operation were 400.1 and 398.7, respectively (*p* = 0.891). However, the mean PEF of the ACBT group (308.7) was significantly higher than that of the control group (254.2) (*p* < 0.001) on POD 7. We also observed that there was no correlation between PEF and daily SWW (correlation coefficient ≈ 0). The mean PaO_2_ (mmHg) of the ACBT group was significantly higher than that of the control group on POD 5 (106.4 vs. 83.00, *p* < 0.001). In the ACBT group, 7.5% of patients required bronchoscopic aspiration, which was less than that required by the control group (15.9%, *p* = 0.02). The median duration of oxygen therapy for the ACBT group (120 h) was less than that for the control group (144 h) (*p* < 0.001). Patient comfort scores (increasing score indicates greater discomfort) were significantly greater for patients in the control group than for those in the ACBT group (*p* < 0.001) **(**Table 4 **)**.

**TABLE 4:** Efficacy and acceptability of techniques for airway clearance

Variable		Time	ACBT	Control	*p* value
Sputum wet weight (g)		BOD 1	2.30(0.48)	2.33(0.48)	0.705
Mean (SD)		POD1	9.60(4.66)	6.88(2.24)	<0.001
		POD2	16.93(3.76)	11.28(2.54)	<0.001
		POD3	24.11(4.54)	14.06(3.60)	<0.001
PaO_2_ (mmHg), mean (SE)		BOD1	88.39(8.83)	87.62(9.40)	0.470
		POD5	106.41(20.19)	89.7(22.66)	<0.001
PEF (L/min), mean (SE)		BOD1	400.1(79.5)	398.7(83.3)	0.891
		POD7	308.7(70.6)	254.2(58.2)	<0.001
O_2_ therapy (h), median (range)			120(24–576)	144(24–1632)	<0.001
Bronchoscopy aspiration, *n* (%)			11(7.5)	23(15.9)	0.029
Patient comfort					
VAS, *n* (%)	0–2		56(38.4)	18(12.4)	<0.001
	3–5		72(49.3)	91(62.8)	
	6–8		18(12.3)	34(23.4)	
	9–10		0(0)	2(1.4)	

Abbreviations: BOD, before operative day; PEF, peak expiratory flow; POD, postoperative day; SD, standard deviation; VAS, visual analogue scale.

## DISCUSSION

To the best of our knowledge, this is the first report of a randomized clinical trial investigating the impact of ACBT and CCP on PPC after esophagectomy. Our results showed that PPC incidence reduced by 50% in the ACBT group when compared with that in the control group. In addition, both incidence of AL and of postoperative hospital LOS, which are important clinical and economical outcomes, significantly decreased in the ACBT group. In the view of these results, ACBT offers a promising chest physiotherapy avenue to reduce PPC after esophagectomy.

PPC is the one of the most common complications seen after esophagectomy. Based on the different definitions and criteria of PPC, its incidence after esophagectomy ranges from 20% to 37% in studies on large datasets or high‐volume single institutions.^3^, ^4^, ^5^, ^6^, ^7^, ^8^ In this study, by adopting the MGS, we noted the incidence of PPC to be 31.0% in the control group, consistent with that reported in other studies.^3^, ^4^, ^5^, ^6^, ^7^, ^8^ In the ACBT group，the incidence of PPC significantly reduced to 15.2%. Using multivariable analysis to control for possible confounders, we found that approximately 60% decreased odds of PPC in the ACBT group. Our analysis suggested that patients who received ACBT were able to effectively clear secretions from their own airways, thus reducing the risk of progressing to PPC due to secretion retention. Current literature on the impact of perioperative chest physiotherapy on PPC after thoracic surgery is limited. A previously published large, quasi‐experimental study demonstrated that the frequency of pulmonary morbidity (pulmonary atelectasis and pneumonia) was 15.5% before initiating a perioperative intensive physiotherapy program and 4.7% after the program (*p* < 0.001) in lung cancer patients after lobectomy.^21^ A randomized clinical trial showed that preoperative intensive inspiratory muscle training reduced the incidence of PPC (18%) in the intervention group as compared to that in the control group (35.5%) for patients undergoing coronary artery bypass graft surgery.^22^ Another prospective single‐blind randomized controlled trial suggested that postoperative incentive spirometry after lobectomy improved the overall recovery of lung function in patients with COPD or smokers. The observed actual difference in the frequency of PPC in favor of the intervention was also larger (14% vs. 23%).^23^ Although these studies vary in terms of the methods of chest physiotherapy and surgical settings, the overall results demonstrate that patients benefit from chest physiotherapy to prevent PPC after thoracic surgery. Hence, we strongly recommend perioperative chest physiotherapy for thoracic surgical patients.^13^

AL is another common complication observed after esophagectomy and is a major cause of morbidity and mortality. The incidence of AL varies from 11% to 20% in large datasets and high‐volume centers.^3^, ^4^, ^6^, ^7^, ^8^, ^9^ In the current study, AL incidence in the control group was 12.4%, in keeping with the findings reported in previous studies.^3^, ^4^, ^6^, ^7^, ^8^, ^9^ Patient characteristics, postoperative factors, and surgical techniques were found to be associated with an increased risk of AL.^24^ In addition, the most important predisposing factors for AL were adequate gastric conduit perfusion to prevent ischemia^25^ and blood oxygenation.^26^ Thus, maintenance of adequate oxygenation during the postoperative period is critical for wound healing.^9^ PPC is also associated with postoperative hypoxemia and hypotension, which are thought to stimulate the release of soluble proinflammatory mediators that impair wound healing.^27^ These consequences could explain the impact of PPC on the occurrence of AL. Michelet et al. reported that development of acute respiratory distress syndrome (ARDS) was a factor that significantly increased AL occurrence.^9^ In this study, we did not investigate the association between PPC and AL, but we did observe that the incidence of both PPC and AL significantly decreased in the ACBT group. We also found that the mean PaO_2_ of the ACBT group, as measured on POD 5, was higher than that of the control group. These findings support the influence of PPCs and maintenance of adequate oxygenation during the postoperative period on the occurrence of AL. Moreover, they demonstrate the protective effect of ACBT on the prevention of AL in patients after esophagectomy.

As an airway clearance technique, variants of ACBT to enhance secretion clearance have been proposed.^16^ Forced expiratory maneuvers (huffing) are reported to promote secretion movement through changes in airway dynamics and thoracic pressure.^14^ Breathing control is thought to prevent bronchospasm and oxygen desaturation, while thoracic expansion exercises aid in loosening and clearance of secretions and improvement of collateral ventilation.^28^ SWW has been used as a reliable and clinically useful outcome of airway clearance techniques.^29^ In a systemic review and meta‐analysis conducted by Lewis et al.,^16^ the standardized mean difference showed an increase in SWW during and up to 1 h post‐ACBT as compared to CCP in patients with chronic lung disease. In the current study, ACBT significantly increased the daily SWW when compared with CCP on POD 1–3. It is not clear whether PEF can accurately assess the efficacy of airway clearance techniques. PEF is mainly considered to reflect large airway function and commonly serves as a global indicator of airway function.^20^ We did not observe any correlation between PEF and daily SWW, in keeping observations from other published studies.^30^ However, the mean PEF was significantly higher in the ACBT group than in the control group on POD 7, suggesting that the degree of airway patency was higher in the ACBT group. These findings demonstrate that ACBT is an effective airway clearance technique in patients after esophagectomy.

In addition to efficacy, the acceptability and tolerability of the airway clearance technique are another important consideration, especially for patients with incision after operation. During CCP, clapping with the hands can vibrate the thorax and facilitate secretion mobilization.^14^ However, clapping may also accentuate postoperative pain and be uncomfortable for the patient. Consequently, patients are reluctant to cough deeply, compromising the efficacy of airway clearance. In contrast, ACBT allows patients to control breathing by themselves without foreign forces on the chest. ACBT has been reported as a well‐tolerated and accepted chest physiotherapy technique in patients with chronic lung disease^14^, ^16^ and in lung cancer patients after lobectomy.^18^ We used a patient comfort score to directly measure the acceptability and tolerability of the treatment from patients' perspective. We found that patients in the control group felt a significantly higher level of discomfort after the intervention than those in the ACBT group. Our results further support the findings of previous reports that ACBT is an airway clearance technique with high acceptability and tolerability.

Our study has several limitations. First, although ACBT achieves a short‐term improvement in sputum clearance, its long‐term outcomes, such as improved pulmonary function and quality of life after esophagectomy, need further investigation. Second, the sample size was large enough to be representative of the postoperative population, but this being a single‐center study, the generalizability of our result is limited. Therefore, a validation of this result should be performed using multicenter studies. Third, standard protocols for chest physiotherapy in perioperative management after thoracic surgery are still absent. Additional work is required to determine the manner in which such treatments should be implemented.^31^ Many questions regarding the optimal daily frequency of therapy, number of days in the perioperative period for which such therapy should be provided, and method of selecting individuals through preoperative risk assessment remain to be answered by further clinical investigations. Hence, the physiotherapy protocols recommended by our study provide only one option for the improvement of current practices.

## CONCLUSIONS

Our study is the first to present ACBT as an effective, well‐tolerated, and accepted airway clearance technique that significantly reduces the incidence of PPC after esophagectomy. ACBT could also significantly reduce both AL and LOS. Its clinical impact on PPC after esophagectomy needs further validation through multicenter studies.

## CONFLICT OF INTEREST

The authors declare no potential conflicts of interest.
